# Relationship Between Floppy Eyelid Syndrome and Obstructive Sleep Apnea Syndrome: An Umbrella Review

**DOI:** 10.1155/joph/6084912

**Published:** 2026-07-14

**Authors:** Gisela Tan Oh Napoli, Daniel Mendes Lobato, Fátima Rosana Albertini, Almiro José Machado Júnior

**Affiliations:** ^1^ Department of Otorhinolaryngology, Head and Neck Surgery, Postgraduate Program in Surgical Sciences, Faculdade de Ciências Médicas (FCM), Universidade Estadual de Campinas (UNICAMP), Campinas, São Paulo, Brazil, unicamp.br; ^2^ Institute of Science and Technology, Universidade Estadual de São Paulo (UNESP), São José dos Campos, São Paulo, Brazil, unesp.br

**Keywords:** eyelid laxity, floppy eyelid syndrome, obstructive sleep apnea

## Abstract

**Background:**

Obstructive sleep apnea syndrome (OSAS) is characterized by intermittent partial or complete interruption of airflow due to upper airway collapse during sleep. Increased eyelid elasticity and the presence of a lax tarsus are features that define floppy eyelid syndrome (FES). The prevalence of FES may reach up to 64.5% among patients with OSAS. Despite its strong association with cardiovascular events, OSAS remains largely underdiagnosed. The *objective* of this study was to evaluate the degree of epidemiological association between FES and OSAS.

**Methods:**

Systematic reviews were evaluated to investigate the association between OSAS and FES in adults. This umbrella review was registered in PROSPERO and followed the PICO framework to define the research question. From August 2023 to January 2025, multiple databases were searched using relevant terms. The methodological quality of the included reviews was assessed using the AMSTAR 2 tool. Certainty of evidence was evaluated using a GRADE framework adapted for umbrella reviews, and an overlap analysis was performed.

**Results:**

Seven systematic reviews were included. According to AMSTAR 2, most were classified as having low methodological confidence. The final GRADE certainty ranged from low to moderate. Overlap analysis indicated moderate overlap. Nevertheless, the included studies consistently reported an association between FES and OSAS.

**Conclusion:**

Consistent with previous literature, this review supports an association between FES and OSAS. Identification of FES or EL during clinical examination may increase suspicion of OSAS. Despite its clinical relevance, this association remains underrecognized, and further studies using standardized diagnostic criteria are needed to strengthen the available evidence.

## 1. Introduction

Episodes of interrupted or shallow breathing during sleep lasting 10 s or longer, resulting from total or partial collapse of the upper airway, characterize obstructive sleep apnea syndrome (OSAS) [1]. Approximately 34% of middle‐aged men and 17% of middle‐aged women meet the diagnostic criteria for OSAS [1]. In a review study, Benjafield et al. [2] estimated that nearly 1 billion individuals worldwide aged 30–69 years may have OSAS, and approximately 425 million individuals have moderate‐to‐severe OSAS for whom treatment is recommended.

Floppy eyelid syndrome (FES) was first described in 1981 by Culbertson and Ostler [3]. It is characterized by eyelid laxity (EL) permitting spontaneous eversion (sometimes occurring during sleep), a malleable tarsus, and chronic papillary conjunctivitis, with these changes typically occurring on the side on which the patient sleeps [3–5]. Common symptoms include nonspecific ocular irritation, mucous discharge, dry eye sensation, photosensitivity, eyelid swelling, and conjunctival redness [6, 7]. Despite being a well‐recognized clinical condition, the diagnostic criteria for FES vary among authors [8]. Diagnosis is based on physical examination combined with patient‐reported symptoms [9].

**FIGURE 1:**
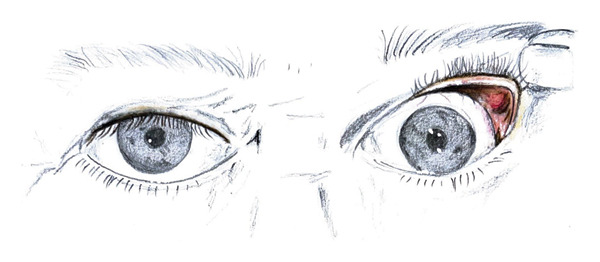
Lash ptosis in the right eye and upper eyelid laxity upon traction during clinical examination of the left eye (illustration created by the author).

**FIGURE 2:**
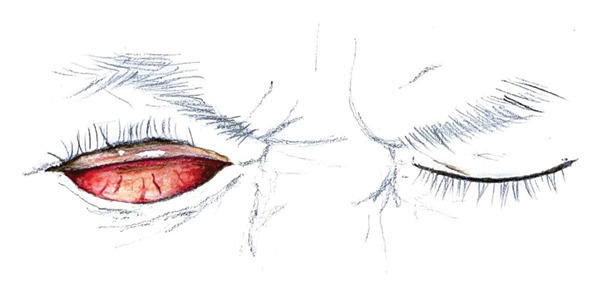
Forceful eyelid closure inducing spontaneous eversion of the right upper eyelid (illustration created by the author).

Other eyelid abnormalities, including eyelid ptosis, eyelash ptosis, blepharochalasis, ectropion, and entropion, may be associated with FES. Corneal alterations ranging from punctate epithelial keratitis to corneal ulcers and infectious keratitis may also occur, with rare cases of ocular perforation reported [7]. Keratoconus, a well‐described corneal ectasia, has also been reported in association with FES [10–12] (Figures 1 and 2).

It has been hypothesized that intermittent hypoxemic episodes caused by OSAS lead to oxidative stress, increased reactive oxygen species production during ischemia–reperfusion injury, and inflammation [13]. These mechanisms may contribute to elevated serum levels and increased matrix metalloproteinase activity in patients with OSAS [14]. Higher concentrations of metalloproteinases have been identified in areas of elastic fiber depletion in patients with FES compared to controls, suggesting that increased elastolytic enzyme activity results in elastic fiber degradation and, consequently, EL and ptosis [15, 16].

The prevalence of FES in the general population ranges from 3.8% to 15.8% [17]. It is commonly associated with male sex, middle age, and a body mass index > 30 kg/m^2^. However, it has also been reported in women (approximately 30% of cases) and, less frequently, in the pediatric population [5]. The prevalence of FES among patients with OSAS has been reported to reach up to 64.5%, with substantial variability across studies [5]. Conversely, the prevalence of FES in individuals diagnosed with OSAS is estimated to range from 4% to 16%, and isolated EL without additional associated symptoms has been linked to OSAS in up to 61% of cases [9].

The prevalence of OSAS ranges from 40% to 80% among patients with hypertension, heart failure, coronary artery disease, pulmonary hypertension, atrial fibrillation, and stroke. Despite its high prevalence in patients with cardiovascular disease and its association with sudden cardiac death, OSAS remains frequently underdiagnosed and undertreated [18, 19].

Although systematic reviews and meta‐analyses have explored the association between FES and OSAS, our search identified no published umbrella reviews addressing this topic. Therefore, the primary objective of this study was to assess the available evidence regarding the degree of association between FES and OSAS.

## 2. Methodology

### 2.1. Protocol and Registration

The Preferred Reporting Items for Overviews of Reviews (PRIOR) Statement was followed in conducting this umbrella review. The study was registered in PROSPERO under the identifier CRD42023430098 [20, 21]. Ethical approval was not required, as this study constitutes an umbrella review of previously published data.

### 2.2. Eligibility Criteria

The PICOS acronym was used to define the eligibility criteria to answer the following question:

“What is the degree of epidemiological association between FES and OSAS?” [22].

• P (Population): Adults (≥ 18 years);
• I (Intervention/Exposure): Patients diagnosed with OSAS;
• C (Comparison): Patients without OSAS or studies without a control group;
• O (Outcome): Prevalence or association between OSAS and FES;
• S (Study type): Systematic reviews, with or without meta‐analysis.

#### 2.2.1. Inclusion and Exclusion Criteria

Systematic reviews, with or without meta‐analyses, involving human subjects and evaluating the prevalence of or association between FES and OSAS were included. No restrictions were applied regarding language or publication date. Literature reviews, expert opinions, in vitro or animal studies, letters, conference abstracts, case reports, and case series were excluded.

### 2.3. Information Sources and Search Strategy

Search strategies were adapted for each electronic database, including Embase, Latin American and Caribbean Health Sciences Literature (LILACS), LIVIVO, PubMed/MEDLINE, Scopus, and Web of Science. Additionally, Google Scholar and ProQuest Dissertation and Theses were searched. On November 16, 2024, searches of the gray literature were conducted concurrently with the database searches. The search was performed on a single day, and no restrictions were applied regarding publication date or language. The adapted search strategies for each database and gray literature source are provided in Appendix 1. Retrieved references were managed using EndNote software (Thomson Reuters, Philadelphia, PA), and duplicate records were removed.

### 2.4. Study Selection and Data Collection Process

The study selection process was conducted in two phases by two independent reviewers (G.T.O.N. and F.R.A.) using the Rayyan Intelligent Systematic Review platform, which allows blinded and independent evaluation.

• Phase 1: Initial screening based on titles and abstracts.
• Phase 2: Full‐text review of selected articles to determine their eligibility. Final inclusion decisions were based on full‐text evaluation.

A calibration phase preceded the study selection to ensure adequate agreement among reviewers. This step involved evaluating 100 references, allowing the calculation of the Kappa agreement coefficient. Phases 1 and 2 commenced only after a Kappa coefficient greater than 0.7 was achieved.

The following data were extracted and recorded for each included study:

• Author; year of publication; country; participant characteristics (sample size and age); outcome measures; results; and relevant conclusions.

### 2.5. Risk‐of‐Bias Assessment

The AMSTAR 2 tool [23] was used to assess the methodological quality of eligible systematic reviews and meta‐analyses. This instrument consists of 16 items evaluating:

• Definition of the research question (PICO);
• Prior protocol registration (e.g., PROSPERO) to ensure transparency;
• Comprehensiveness of search strategy and study selection, including dual review;
• Assessment of risk of bias in included studies;
• Use of appropriate statistical methods (including meta‐analysis, when applicable);
• Consideration of publication bias;
• Discussion of limitations within the systematic review.

According to AMSTAR 2, overall confidence is categorized as follows:

• High: No or only one noncritical weakness;
• Moderate: More than one noncritical weakness;
• Low: One serious flaw, with or without additional weaknesses;
• Very low: More than one serious flaw.

Classification accuracy was ensured using the AMSTAR 2 platform to determine overall methodological confidence.

### 2.6. Certainty of Evidence

The certainty of the body of evidence regarding the association between FES and OSAS was assessed using the Grading of Recommendations Assessment, Development, and Evaluation (GRADE) [24] framework, adapted for umbrella reviews. It is important to emphasize that GRADE evaluates certainty in the effect estimate, whereas AMSTAR 2 evaluates the methodological rigor of the systematic review process.

GRADE categorizes certainty into four levels: high, moderate, low, and very low. Certainty was downgraded by one or two levels in the presence of serious methodological limitations (risk of bias), inconsistency (substantial statistical heterogeneity), indirectness, imprecision of confidence intervals, or publication bias.

Conversely, certainty was upgraded when a large magnitude of effect was observed, when evidence of a biological dose–response gradient was present, or when residual confounding was rigorously addressed. The final certainty was categorized as high, moderate, low, or very low.

### 2.7. Overlap Analysis

To avoid artificial inflation of evidence due to overlapping primary data across multiple meta‐analyses, an assessment of primary study overlap was performed. Primary studies from each systematic review were independently extracted and compiled into an evidence matrix to identify unique and duplicate publications.

Data redundancy was quantified using the corrected covered area (CCA) index [25], which allows classification of the degree of overlap and assessment of whether the current synthesis incorporates novel data or primarily reiterates previously consolidated evidence. The CCA was interpreted according to established thresholds to classify the degree of overlap as slight, moderate, high, or very high [26].

## 3. Results

### 3.1. Study Selection

A total of 685 studies were identified through database searches, of which 677 were excluded following title and abstract screening. From the gray literature, 125 articles were retrieved, of which 119 were excluded. After duplicate removal, nine studies remained for full‐text assessment; two were excluded because they were narrative literature reviews (Appendix 2).

**FIGURE 3:**
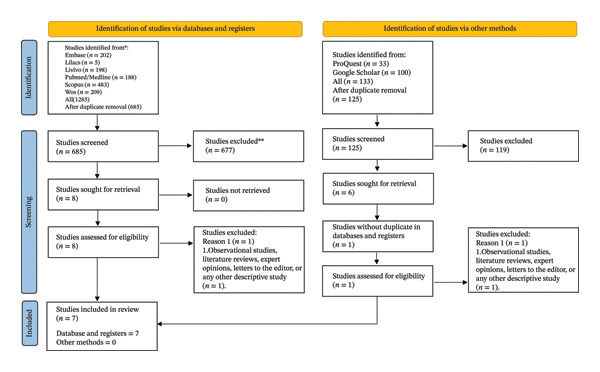
PRISMA flow diagram adapted according to PRIOR recommendations.

Seven studies were included by two independent reviewers (G.T.O.N. and F.R.A.), as illustrated in Figure 3.

### 3.2. Characteristics of the Included Systematic Reviews

The included systematic reviews [27–33] comprised various observational study designs, including case–control, cross‐sectional, and cohort designs. All assessed the association between FES and OSAS. Some reviews also investigated additional ophthalmologic manifestations associated with OSAS [28, 30, 33].

The reviews included between 6 and 17 primary studies. Study populations ranged from 511 to 1865 participants, predominantly male, with ages ranging across reviews from 38 to 62.7 years.

Polysomnography was the primary diagnostic tool for OSAS across studies. Some reviews also included studies that used validated questionnaires [27, 30, 31, 33] or nocturnal oximetry [31, 32]. OSAS severity was assessed in all studies through the apnea–hypopnea index (AHI).

### 3.3. Summary of Results

Table 1 summarizes the findings of the seven systematic reviews [27–33] included in this umbrella review.

**TABLE 1:** Characteristics of included studies.

Author	Country	Objectives	Total studies included	Study population	Outcomes assessed	Diagnostic instruments	Mention of items: 1 PRISMA 2 PROSPERO 3 Meta‐analysis	Financing	Main results
Bulloch et al., 2023	Australia	To assess the prevalence of OSAS in FES	*N* = 12 Cross‐sectional = 4 Retrospective = 7 Case–control = 1	Adult Average age—55.1 years Male—77.6% Total—511 FES—475 OSAS—288	Prevalence of OSAS in SFP, severity of the association.	PSG and validated questionnaires	1 yes 2 yes 3 yes	None	The overall prevalence of OSAS in FES was 57.1%. (95% CI: 46.5%–74.8%)

Huon et al., 2016	USA	To assess the association between OSA and ophthalmological pathologies	*N* = 7 Cross‐sectional = 7	Adult Average age—NA Male—NA Total—902 FES—312 OSAS—690	Association of OSAS and ophthalmological diseases.	PSG and AHI	1 yes 2 no 3 yes	None	The overall pooled OR for FES was 3.126 (*p* < 0.001)

Aiello et al., 2023	Italy	Evaluate the association between EL and FES at OSAS	*N* = 11 Cross‐sectional = 8 Case–control = 2 Case series = 1	Adult average age—47–54 years (EL); 39.2–56.8 (FES) Male—NA Total—584 FES—153 EL—431 OSAS—517	To assess the degree of prevalence of EL and FES in OSAS, risk of EL and FES in OSAS, and distinct criteria for EL and FES.	PSG and AHI	1 yes 2 yes 3 yes	Yes (Universita degli Studi di Roma Tor Vergata)	The prevalence of EL was 40.2% (95% CI 28.6%–53.1%) and FES 22.4% (95% CI: 13.8%–34.2%) in OSAS. OSAS has a risk of 3.4 times (95% CI: 2.2–5.2) for EL and 3.0 times (95% CI: 1.7–5.5) for FES.

Bulloch et al., 2024	Australia	Evaluate the association between OSAS and eye diseases	*N* = 6 Cross‐sectional = 3 Case–control = 3	Adult average age—NA Male—NA Total—920 FES—659 OSAS—NA	Prevalence of OSAS in the presence of FES. Presence of other pathologies (ocular and systemic) associated with FES.	PSG, AHI, and validated questionnaires	1 yes 2 yes 3 yes	None	The OR of FES in OSAS was 3.68 (95% CI 2.18–6.20). There was a significant association of OSAS with NOINA, FES, RVO, KC, CRS, and glaucoma

Cheong et al., 2023	Singapore	Evaluate the association between OSAS and FES	*N* = 15 Prospective cohort = 3 Cross‐sectional = 10 Case–control = 2	Adult average age‐40–59.6 Male—47%–83% Total—1865 FES—NA OSAS—NA	Association of OSAS and FES, association of OSAS severity and FES, CPAP treatment in OSAS and FES.	PSG, AHI, oximetry, and validated questionnaires	1 yes 2 yes 3 yes	None	The OR of FES in OSAS was 1.89 (95% CI: 1.27–2.83), Severe OSAS OR of 3.51, (95% CI = 2.03–6.07), moderate OR 2.85 (95% CI 1.60–5.07) and slight OR 2.24 (95% CI = 1.22–1.40)

Wang et al., 2016	China	To assess if FES is more prevalent in OSAS	*N* = 6 Case series = 1 Cross‐sectional = 4 Case–control = 1	Adult average age—43.2–62.7 Male—549 Total—767 FES—259 OSAS—609	Prevalence of FES in OSAS and whether the severity of OSAS has a greater association with FES.	PSG and AHI or oximetry	1 yes 2 no 3 yes	None	The OR of FES in OSAS was 4.12 (95% CI 2.36‐7.20). Higher incidence of FES with OSAS severity: mild OR = 2.56 (1.05–6.28); moderate OR = 4.62 (1.99–10.73); severe OR = 7.64 (3.44–16.96)

Sun et al., 2023	China	To determine whether changes in the ocular surface, including FES, are associated with more severe OSAS	*N* = 6 Case–control = 2 Cross‐sectional = 4	Adult average age—38–52.6 Male—535 Total—NA FES—286 OSAS—627	Prevalence of FES in OSAS, dry eye syndrome in OSAS.	STOP‐BANG questionnaire, PSG and AHI	1 yes 2 yes 3 yes	None	FES in OSAS was 40% (95% CI 0.37–0.43; *p* < 0.001).

*Note:* OSAS, obstructive sleep apnea; NOINA, non–arteritic ischemic optic neuritis.

Abbreviations: AHI, apnea–hypopnea index; CPAP, continuous positive airway pressure; CRS, central serous retinopathy; EL, eyelid laxity; FES, floppy eyelid syndrome; NA, not available; OR, odds ratio; PSG, polysomnography; RVO, retinal vein occlusion.

Bulloch et al. [27] reported a pooled prevalence of OSAS among patients with FES of 57.1%. This meta‐analysis included 12 observational studies comprising 511 patients with FES, predominantly middle‐aged men (77.6%), with a pooled mean age of 55.1 years, and approximately 69% of whom received a new diagnosis of OSAS during the study. The diagnosis of OSAS across included studies was primarily established using polysomnography and validated sleep questionnaires, whereas FES was generally defined by upper eyelid hyperlaxity and easy eyelid eversion.

Huon et al. [28] demonstrated a strong association between OSAS and FES, reporting a pooled OR of 3.126. This systematic review and meta‐analysis included seven observational studies, evaluating the association between OSAS and FES, involving 690 patients with OSAS. Polysomnography and the Epworth Sleep Questionnaire were used as diagnostic tools for OSAS. FES diagnosis was also variable, ranging from subjective EL and easy eversion to papillary conjunctivitis and ocular irritation.

Aiello et al. [29] review included 11 prospective observational studies involving 1225 adult patients with OSAS and differentiated isolated EL from FES. Among these patients, 431 presented with EL and 153 with FES. The pooled prevalence of FES was 22.4%, and for EL was 40.2% among patients with OSAS. OSAS patients showed approximately a threefold increased risk of developing either FES or EL compared with controls. In this review, OSAS was mainly diagnosed by polysomnography and AHI criteria, while FES was defined as eyelid hyperlaxity associated with chronic papillary conjunctivitis, and EL was characterized by isolated EL without inflammatory findings.

Bulloch et al. [30] further confirmed the significant association between OSAS and FES, reporting a pooled OR of 3.68. This review included six observational studies that analyzed adult patients with OSAS and matched control groups. FES prevalence in OSAS patients ranged from 25.8% to 64.6%, with participants predominantly consisting of middle‐aged, overweight, or obese individuals. OSAS diagnosis relied predominantly on polysomnography, AHI thresholds, and clinical criteria, whereas FES was diagnosed clinically based on eyelid hyperlaxity and easy eversion, associated with chronic conjunctival changes.

Cheong et al. [31] also demonstrated a positive association between OSAS and FES, with pooled ORs ranging from 1.89 to 2.10. 15 observational studies, comprising 1865 adult patients, predominantly middle‐aged, overweight, or obese individuals, were included. A severity‐dependent relationship was identified: increasing OSAS severity correlated with progressively greater FES risk. The diagnostic criteria across studies included polysomnography, AHI classifications, the Epworth sleep questionnaire, and the STOP‐BANG questionnaire, while FES diagnosis relied on eyelid hyperlaxity, papillary conjunctivitis, and eyelid eversion findings.

Similarly, Wang et al. [32] reported a pooled OR of 4.12 for FES prevalence among patients with OSAS. Six observational studies were included in this meta‐analysis. The pooled population comprised 767 participants, predominantly middle‐aged men. A progressive increase in FES prevalence with increasing OSAS severity was observed, with ORs ranging from 2.56 to 7.64 across mild‐to‐severe OSAS. OSAS diagnosis was established using polysomnography, oxygen desaturation index, respiratory disturbance index, and AHI criteria, whereas FES diagnosis was primarily based on subjective clinical findings, including easy eyelid eversion, eyelid hyperlaxity, and papillary conjunctivitis.

Sun et al. [33] reported a pooled prevalence of FES of approximately 40% among patients with OSAS. This meta‐analysis included six observational studies comprising 627 adult patients with OSAS, of whom 286 had FES, and predominantly consisted of middle‐aged male patients. OSAS diagnosis was predominantly established through polysomnography and AHI classification. FES was generally diagnosed clinically based on eyelid hyperlaxity and easy eyelid eversion.

Across reviews, reported odds ratios ranged from 1.89 to 7.64, indicating a positive association between FES and OSAS. Higher OSAS severity was generally associated with a greater likelihood of FES [31, 32].

**FIGURE 4:**
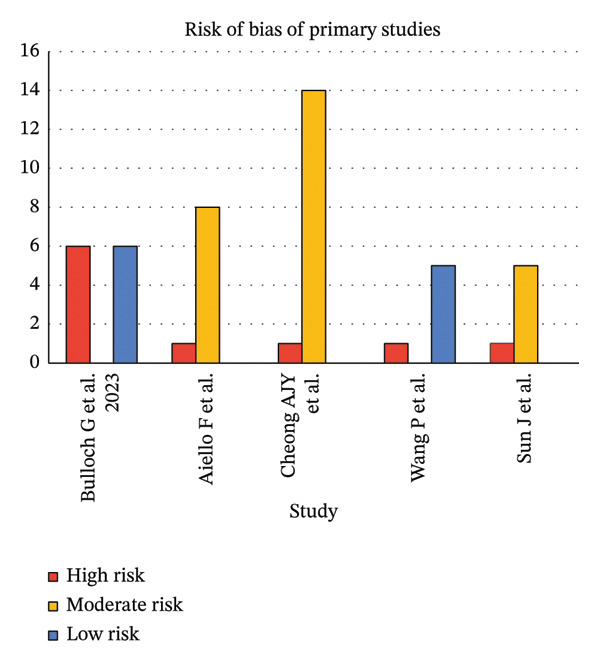
Risk of bias of primary studies.

Heterogeneity was high in three reviews [27, 29, 33], moderate in one review [31], and low in two reviews [30, 32]. The primary studies included in these reviews were predominantly of moderate methodological quality (Figure 4 and Table 2).

**TABLE 2:** Heterogeneity and risk of bias of primary studies.

Study	Heterogeneity	Tool for assessing risk of bias	Quality of primary studies
Bulloch et al., 2023	High (*I* ^2^ = 87.8%)	JBI‐PCAT	High risk 6 low risk 6
Huon et al.	NA	NA	Na
Aiello et al.	High (*I* ^2^ = 78%)	JBI‐PCAT	High risk 1 Moderate risk 8
Bulloch et al., 2024	Low (*I* ^2^ = 29%)	NA	Na
Cheong et al.	Moderate (*I* ^2^ = 42%)	Newcastle–Ottawa	Alto risk 1 Moderate and low risk 14
Wang et al.	Low (*I* ^2^ = 21%)	Own scale	Low quality 1 High quality 5
Sun et al.	Low (*I* ^2^ = 95.8%)	Newcastle–Ottawa	High risk 1 Moderate risk 5

Abbreviations: JBI, PCAT, Joanna Briggs Institute Prevalence Critical Appraisal Tool; NA, not available.

### 3.4. Risk of Bias in Systematic Reviews

Using the AMSTAR 2 tool to assess methodological quality, four studies were classified as having low overall confidence, whereas the remaining studies were classified as critically low.

**FIGURE 5:**
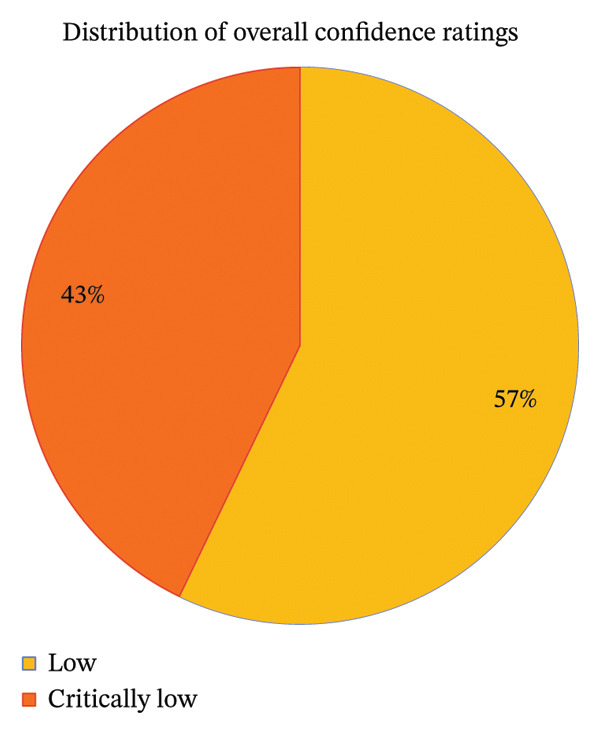
Distribution of overall confidence ratings.

The following factors clarify this low overall score: two reviews [28, 32] were not registered in PROSPERO, none provided a detailed list of excluded primary studies; three reviews [28, 30, 32] did not include a formal risk‐of‐bias assessment; one review [30] did not consider risk of bias in its meta‐analysis discussion; and two reviews [28, 30] did not assess publication bias. These findings are summarized in Table 3 and Figure 5.

**TABLE 3:** AMSTAR 2 assessment of included studies.

Study	1	2^∗^	3	4^∗^	5	6	7^∗^	8	9^∗^	10	11^∗^	12	13^∗^	14	15^∗^	16	General confidence
Bulloch et al., (2023)	Yes	Yes	Yes	Yes	Yes	Yes	No	Yes	Yes	No	Yes	Yes	Yes	Yes	Yes	Yes	Low
Huon et al., (2016)	Yes	No	Yes	Yes	Yes	Yes	No	Yes	No	No	Yes	No	Yes	Yes	No	Yes	critically low
Aiello et al., (2023)	Yes	Yes	Yes	Yes	Yes	Yes	No	Yes	Yes	No	Yes	Yes	Yes	Yes	Yes	Yes	Low
Bulloch et al., (2024)	Yes	Yes	Yes	Yes	Yes	Yes	No	Yes	No	No	Yes	No	No	Yes	No	Yes	critically low
Cheong et al., (2023)	Yes	Yes	Yes	Yes	Yes	Yes	No	Yes	Yes	No	Yes	Yes	Yes	Yes	Yes	Yes	Low
Wang et al., (2016)	Yes	No	Yes	Yes	Yes	Yes	No	Yes	Partially Yes	No	Yes	No	Yes	Yes	Yes	Yes	critically low
Sun J. et al. (2023)	Yes	Yes	Yes	Yes	Yes	Yes	No	Yes	Yes	No	Yes	No	Yes	Yes	Yes	Yes	Low

*Note:* Items with an asterisk are considered critical [23].

### 3.5. Certainty of Evidence

The seven included systematic reviews demonstrated significant associations between OSAS and various ocular pathologies. Within the GRADE framework, large effect sizes may justify upgrading certainty.

FES was the ocular condition most consistently associated with OSAS. Patients with OSAS exhibited an increased risk of FES (or its precursor condition, EL), with reported odds ratios ranging from 1.89 to 4.69.

According to the GRADE framework, adapted for umbrella reviews, and given that all included studies were observational (cross‐sectional and case–control), the initial certainty rating for each outcome was low [24]. Any downgrading or upgrading was performed in accordance with predefined criteria, as detailed in Table 4.

**TABLE 4:** Adapted GRADE analysis of the evidence (association between FES and OSAS).

Author (Year)	Initial level	Risk of bias	Heterogeneity	Indirectness	Imprecision	Publication bias	Upgrading factors	Final certainty (GRADE)
Bulloch et al. (2023)	Low	Downgraded (−1)	Downgraded (−1)	No serious concerns	No serious concerns	Not detected	None	Very low
Huon et al. (2016)	Low	Downgraded (−1)	Downgraded (−1)	No serious concerns	No serious concerns	Not assessed	None	Low
Aiello et al. (2023)	Low	No serious concerns	Downgraded (−1)	No serious concerns	No serious concerns	Not detected	None	Low
Bulloch et al. (2024)	Low	No serious concerns	No serious concerns	No serious concerns	No serious concerns	Not detected	None	Moderate
Cheong et al. (2023)	Low	No serious concerns	No serious concerns	No serious concerns	No serious concerns	Not detected	Dose–response (+1)	Moderate
Wang et al. (2016)	Low	No serious concerns	No serious concerns	No serious concerns	No serious concerns	Suspected	Large magnitude of effect (+1)	Moderate
Sun J et al. (2023)	Low	No serious concerns	Downgraded (−1)	No serious concerns	No serious concerns	Not detected	None	Low

### 3.6. Overlap Analysis

The overlap analysis identified 63 primary study citations across the seven included systematic reviews. Table 5 presents the list of primary studies extracted from each review, focusing on FES/EL outcomes.

**TABLE 5:** Extraction of primary studies.

Systematic review	Total studies	List of extracted primary studies
Bulloch et al. (2023)	12	Tyagi (2018), Compton (2016), Oral (2010), Yeung (2014), Netland (1994), Sward (2018), NKM (2020), Taban & Perry (2006), Ezra (2010), Schlötzer‐Schrehardt (2005), Muniesa (2013), Fowler (2010)
Huon et al. (2016)	7	Mojon (1999), Karger (2006), Kadyan (2010), Beis (2012), Chambe (2012), Acar (2013), Muniesa (2013)
Aiello et al. (2023)	11	Cristescu (2020), Sward (2018), Acar (2013), Muniesa (2013), Muniesa (2014), Chambe (2012), Beis (2012), Kadyan (2010), Karger (2006), Robert (1997), Mojon (1999)
Bulloch et al. (2024)	6	Karger (2006), Ezra (2010), Kadyan (2010), Beis (2012), Chambe (2012), Acar (2013)
Cheong et al. (2023)	15	Acar (2013), Acar (2014), Beis (2012), Chambe (2012), Ezra (2010), Folsom (2022), Karger (2006), Kadyan (2010), Lin (2022), Mohamed (2014), Muniesa (2013), Muniesa (2014), Nijjar (2022), Ulutas (2022), Vieira (2020)
Wang et al. (2016)	6	Mojon (1999), Karger (2006), Kadyan (2010), Chambe (2012), Acar (2013), Muniesa Royo (2013)
Sun et al. (2023)	6	Ulutas (2022), Lin PW (2022), Muhafiz (2020), Karaca (2019), Acar (2013), Liu SH (2022)

Using the overlap matrix (evidence matrix; Table 6), which visually represents the intersections between included systematic reviews and their corresponding primary studies, overlap was quantified with the CCA index (Equation (1)) [25].

**TABLE 6:** Evidence matrix: overlap of primary studies (FES–OSAS association).

Primary study	Bulloch (2023)	Huon (2016)	Aiello (2023)	Bulloch (2024)	Cheong (2023)	Wang (2016)	Sun J (2023)	Total
Acar (2013)	X	X	X	X	X	X	X	7
Kadyan (2010)		X	X	X	X	X		5
Karger (2006)		X	X	X	X	X		5
Chambe (2012)		X	X	X	X	X		5
Muniesa (2013)	X	X	X		X	X		5
Beis (2012)		X	X	X	X			4
Ezra (2010)	X			X	X			3
Mojon (1999)		X	X			X		3
Muniesa (2014)			X		X			2
Lin (2022)					X		X	2
Ulutas (2022)					X		X	2
Sward (2018)	X		X					2
Cristescu (2020)			X				X	2
Muhafiz (2020)							X	1
Karaca (2019)							X	1
Liu (2022)							X	1
Mohamed (2014)					X			1
Nijjar (2022)					X			1
Vieira (2020)					X			1
Folsom (2022)					X			1
Acar (2014)					X			1
Tyagi (2018)	X							1
Compton (2016)	X							1
Oral (2010)	X							1
Yeung (2014)	X							1
Netland (1994)	X							1
NKM (2020)	X							1
Taban (2006)	X							1
Schlötzer (2005)	X							1
Fowler (2010)	X							1
Robert (1997)			X					1
Total (N)	12	7	11	6	15	6	6	63

After standardization and removal of duplicate entries across reviews, 31 unique primary studies were identified. The study by Acar et al. was included in all seven reviews. Four additional studies (Kadyan 2010; Karger 2006; Chambe 2012; and Muniesa 2013) constituted the core evidence base, each appearing in five of the seven reviews. The CCA was 0.17 (Equation (1)).

Equation (1) shows CCA calculation [25].

(1) CCA=N−rr×k−1,

*N* = total number of study entries (63), *r* = number of unique primary studies (31), and *k* = number of systematic reviews (7).

## 4. Discussion

This umbrella review included seven systematic reviews with meta‐analyses of observational studies, synthesizing current evidence on the relationship between FES and OSAS. Three reviews [28, 30, 33] also examined additional ophthalmologic conditions alongside FES, further supporting a link between ocular conditions and OSAS [34].

The included reviews reinforce the clinical relevance of FES as a potential ophthalmologic marker of OSAS [35].

Consistent with previous literature [35, 36], the included reviews reported a positive association between FES and OSAS, with odds ratios ranging from 1.89 [31] to 4.12 [32]. A stronger association was observed with increasing OSAS severity [31, 32], contradicting some earlier findings [37] but supporting others [38, 39].

Demonstrating this association may heighten clinical suspicion for OSAS in patients presenting with EL or FES, potentially improving detection of a frequently underdiagnosed condition [40, 41] with important cardiovascular implications [42].

High heterogeneity likely reflects differences in study populations and the absence of standardized diagnostic criteria for both OSAS and FES. Nevertheless, the association remained consistent across reviews, particularly in studies using objective polysomnographic severity criteria.

Most reviews performed formal risk‐of‐bias assessments; however, AMSTAR 2 confidence ratings remained predominantly low or critically low, mainly due to a lack of protocol registration and the absence of excluded‐study lists (Table 3).

Overall certainty of evidence ranged from low to moderate, with upgrades in some reviews due to lower heterogeneity and evidence of a biological gradient [24].

The overlap analysis indicated a moderate overlap among reviews (CCA = 0.17), indicating that a core set of primary studies contributed to multiple reviews [25]. Although this redundancy reinforces the direction of the association, it may also perpetuate similar methodological limitations and biases across evidence syntheses.

Obesity and age are critical confounding factors, as both are independently associated with OSAS and FES. This complicates causal interpretation, as the observed association may partly reflect shared risk factors rather than a direct pathophysiological link [3, 6, 8].

Evidence suggests that the association between OSAS and FES is stronger in cases of severe OSAS confirmed by polysomnography. Severe disease is consistently associated with higher odds of FES, supporting a dose–response relationship. Objective severity criteria likely reduce misclassification and reveal a more robust association between severe OSAS and FES [43, 44].

This umbrella review has inherent limitations because it relies solely on findings from existing systematic reviews and meta‐analyses, and the included evidence is predominantly observational and heterogeneous. Nevertheless, umbrella reviews provide a comprehensive synthesis of available evidence and are particularly useful for determining whether existing literature is consistent or conflicting and for exploring the reasons underlying these patterns [45].

## 5. Conclusion

Consistent with previous literature, this umbrella review supports an association between FES and OSAS. Identification of FES or EL during clinical examination, particularly through recognition of characteristic features and EL, may increase suspicion of OSAS. Despite its clinical relevance, this association remains underrecognized, and further studies using standardized diagnostic criteria are needed to strengthen the available evidence.

## Funding

The authors received no financial support for the research, authorship, and/or publication of this article.

## Conflicts of Interest

The authors declare no conflicts of interest.

## Supporting Information

Additional supporting information can be found online in the Supporting Information section.

## Supporting information


**Supporting Information 1** Appendix 1—Detailed database search strategies used for all electronic databases and gray literature sources cited in Section 2.3.

**Supporting Information 2** Appendix 2—List of excluded full‐text articles and reasons for exclusion, cited in Section 3.1.

## Data Availability

Data sharing is not applicable to this article as no datasets were generated or analyzed during the current study.
