# Supplementary material for: Relationship Between Floppy Eyelid Syndrome and Obstructive Sleep Apnea Syndrome: An Umbrella Review
Source: J Ophthalmol. 2026 Jul 14;2026:6084912. doi: 10.1155/joph/6084912 (PMC13366405; doi:10.1155/joph/6084912)
Supplement: Supplementary file 1 — Supporting Information 1 Appendix 1—Detailed database search strategies used for all electronic databases and gray literature sources cited in Section 2.3. [file JOPH-2026-6084912-s001.docx]

**Appendix 1 -** Database search strategy.

| Database | Search |
| --- | --- |
| PubMed | (“floppy eyelid syndrome” OR “floppy eyelid” OR “eyelid laxity” OR “lax eyelid” OR “FES” OR ”ocular manifestations” OR “Eye manifestations“[MeSH] OR “Eye manifestation“ OR “ophthalmic manifestations“ OR “ophthalmic manifestation“ OR “ocular pathology“ OR “ocular disorders“ OR “ocular disorder“ OR “eye disorders“ OR “eye disorder“ OR “ophthalmic disorder“ OR “ophthalmic disorders“ OR “ophthalmic disease“ OR “ocular disease“ OR “eye disease“ OR “ophthalmic diseases“ OR “ocular diseases“ OR “eye diseases“ OR “ophthalmologic diseases“ OR “ophthalmologic disease“) AND (“Apnea“[MeSH] OR “Apneas“ OR “Sleep Apnea Syndromes“[MeSH] OR “Sleep Apnea Syndrome“ OR “Sleep Apnea“ OR “Sleep Apneas“ OR “Sleep Hypopnea“ OR “Sleep Hypopneas“ OR “Hypersomnia with Periodic Respiration“ OR “Mixed Central and Obstructive Sleep Apnea“ OR “Mixed Sleep Apnea“ OR “Mixed Sleep Apneas“ OR “Sleep-Disordered Breathing“ OR “Sleep Disordered Breathing“ OR “Obstructive Sleep Apnea” OR “Obstructive Sleep Apnea Syndrome” OR “Obstructive Sleep Apneas” OR “OSAHS” OR “Upper Airway Resistance Sleep Apnea Syndrome”) |
| SCOPUS | TITLE-ABS-KEY(“floppy eyelid syndrome” OR “floppy eyelid” OR “eyelid laxity” OR “lax eyelid” OR “FES” OR ”ocular manifestations” OR “Eye manifestations“ OR “Eye manifestation“ OR “ophthalmic manifestations“ OR “ophthalmic manifestation“ OR “ocular pathology“ OR “ocular disorders“ OR “ocular disorder“ OR “eye disorders“ OR “eye disorder“ OR “ophthalmic disorder“ OR “ophthalmic disorders“ OR “ophthalmic disease“ OR “ocular disease“ OR “eye disease“ OR “ophthalmic diseases“ OR “ocular diseases“ OR “eye diseases“ OR “ophthalmologic diseases“ OR “ophthalmologic disease“) AND TITLE-ABS-KEY(“Apnea“ OR “Apneas“ OR “Sleep Apnea Syndromes“ OR “Sleep Apnea Syndrome“ OR “Sleep Apnea“ OR “Sleep Apneas“ OR “Sleep Hypopnea“ OR “Sleep Hypopneas“ OR “Hypersomnia with Periodic Respiration“ OR “Mixed Central and Obstructive Sleep Apnea“ OR “Mixed Sleep Apnea“ OR “Mixed Sleep Apneas“ OR “Sleep-Disordered Breathing“ OR “Sleep Disordered Breathing“ OR “Obstructive Sleep Apnea” OR “Obstructive Sleep Apnea Syndrome” OR “Obstructive Sleep Apneas” OR “OSAHS” OR “Upper Airway Resistance Sleep Apnea Syndrome”) |
| EMBASE | ('floppy eyelid syndrome':kw,ab,ti OR 'floppy eyelid':kw,ab,ti OR 'eyelid laxity':kw,ab,ti OR 'lax eyelid':kw,ab,ti OR 'fes':kw,ab,ti OR 'ocular manifestations':kw,ab,ti OR 'eye manifestations':kw,ab,ti OR 'eye manifestation':kw,ab,ti OR 'ophthalmic manifestations':kw,ab,ti OR 'ophthalmic manifestation':kw,ab,ti OR 'ocular pathology':kw,ab,ti OR 'ocular disorders':kw,ab,ti OR 'ocular disorder':kw,ab,ti OR 'eye disorders':kw,ab,ti OR 'eye disorder':kw,ab,ti OR 'ophthalmic disorder':kw,ab,ti OR 'ophthalmic disorders':kw,ab,ti OR 'ophthalmic disease':kw,ab,ti OR 'ocular disease':kw,ab,ti OR 'eye disease':kw,ab,ti OR 'ophthalmic diseases':kw,ab,ti OR 'ocular diseases':kw,ab,ti OR 'eye diseases':kw,ab,ti OR 'ophthalmologic diseases':kw,ab,ti OR 'ophthalmologic disease':kw,ab,ti) AND ('apnea':kw,ab,ti OR 'apneas':kw,ab,ti OR 'sleep apnea syndromes':kw,ab,ti OR 'sleep apnea syndrome':kw,ab,ti OR 'sleep apnea':kw,ab,ti OR 'sleep apneas':kw,ab,ti OR 'sleep hypopnea':kw,ab,ti OR 'sleep hypopneas':kw,ab,ti OR 'hypersomnia with periodic respiration':kw,ab,ti OR 'mixed central and obstructive sleep apnea':kw,ab,ti OR 'mixed sleep apnea':kw,ab,ti OR 'mixed sleep apneas':kw,ab,ti OR 'sleep-disordered breathing':kw,ab,ti OR 'sleep disordered breathing':kw,ab,ti OR 'obstructive sleep apnea':kw,ab,ti OR 'obstructive sleep apnea syndrome':kw,ab,ti OR 'obstructive sleep apneas':kw,ab,ti OR 'osahs':kw,ab,ti OR 'upper airway resistance sleep apnea syndrome':kw,ab,ti) |
| WEB OF SCIENCE | (“floppy eyelid syndrome” OR “floppy eyelid” OR “eyelid laxity” OR “lax eyelid” OR “FES” OR ”ocular manifestations” OR “Eye manifestations“ OR “Eye manifestation“ OR “ophthalmic manifestations“ OR “ophthalmic manifestation“ OR “ocular pathology“ OR “ocular disorders“ OR “ocular disorder“ OR “eye disorders“ OR “eye disorder“ OR “ophthalmic disorder“ OR “ophthalmic disorders“ OR “ophthalmic disease“ OR “ocular disease“ OR “eye disease“ OR “ophthalmic diseases“ OR “ocular diseases“ OR “eye diseases“ OR “ophthalmologic diseases“ OR “ophthalmologic disease“) AND (“Apnea“ OR “Apneas“ OR “Sleep Apnea Syndromes“ OR “Sleep Apnea Syndrome“ OR “Sleep Apnea“ OR “Sleep Apneas“ OR “Sleep Hypopnea“ OR “Sleep Hypopneas“ OR “Hypersomnia with Periodic Respiration“ OR “Mixed Central and Obstructive Sleep Apnea“ OR “Mixed Sleep Apnea“ OR “Mixed Sleep Apneas“ OR “Sleep-Disordered Breathing“ OR “Sleep Disordered Breathing“ OR “Obstructive Sleep Apnea” OR “Obstructive Sleep Apnea Syndrome” OR “Obstructive Sleep Apneas” OR “OSAHS” OR “Upper Airway Resistance Sleep Apnea Syndrome”) |
| LILACS | ("floppy eyelid syndrome" OR "floppy eyelid" OR "eyelid laxity" OR "lax eyelid" OR "FES" OR "ocular manifestations" OR "Eye manifestations" OR "Eye manifestation" OR "ophthalmic manifestations" OR "ophthalmic manifestation" OR "ocular pathology" OR "ocular disorders" OR "ocular disorder" OR "eye disorders" OR "eye disorder" OR "ophthalmic disorder" OR "ophthalmic disorders" OR "ophthalmic disease" OR "ocular disease" OR "eye disease" OR "ophthalmic diseases" OR "ocular diseases" OR "eye diseases" OR "ophthalmologic diseases" OR "ophthalmologic disease" OR "síndrome del párpado laxo" OR "párpado laxo" OR "laxitud del párpado" OR "párpado flácido" OR "manifestaciones oculares" OR "manifestación ocular" OR "patología ocular" OR "trastornos oculares" OR "trastorno ocular" OR "enfermedad ocular" OR "enfermedades oculares" OR "enfermedades oftálmicas" OR "enfermedad oftálmica" OR "síndrome da pálpebra flácida" OR "pálpebra flácida" OR "laxidade palpebral" OR "manifestação ocular" OR "manifestações oculares" OR "patologia ocular" OR "transtornos oculares" OR "transtorno ocular" OR "doença ocular" OR "doenças oculares" OR "doenças oftálmicas" OR "doença oftálmica") AND ("Apnea" OR "Apneas" OR "Sleep Apnea Syndromes" OR "Sleep Apnea Syndrome" OR "Sleep Apnea" OR "Sleep Apneas" OR "Sleep Hypopnea" OR "Sleep Hypopneas" OR "Hypersomnia with Periodic Respiration" OR "Mixed Central and Obstructive Sleep Apnea" OR "Mixed Sleep Apnea" OR "Mixed Sleep Apneas" OR "Sleep-Disordered Breathing" OR "Sleep Disordered Breathing" OR "Obstructive Sleep Apnea" OR "Obstructive Sleep Apnea Syndrome" OR "Obstructive Sleep Apneas" OR "OSAHS" OR "Upper Airway Resistance Sleep Apnea Syndrome" OR "Apnea del sueño" OR "Apneas del sueño" OR "Síndromes de apnea del sueño" OR "Síndrome de apnea del sueño" OR "Apnea obstructiva del sueño" OR "Síndrome de apnea obstructiva del sueño" OR "Apneas obstructivas del sueño" OR "Trastornos respiratorios del sueño" OR "Apneia do sono" OR "Apneias do sono" OR "Síndrome de apneia do sono" OR "Síndrome de apneia obstrutiva do sono" OR "Apneias obstrutivas do sono" OR "Distúrbios respiratórios do sono") |
| LIVIVO | (“floppy eyelid syndrome” OR “floppy eyelid” OR “eyelid laxity” OR “lax eyelid” OR “FES” OR ”ocular manifestations” OR “Eye manifestations“ OR “Eye manifestation“ OR “ophthalmic manifestations“ OR “ophthalmic manifestation“ OR “ocular pathology“ OR “ocular disorders“ OR “ocular disorder“ OR “eye disorders“ OR “eye disorder“ OR “ophthalmic disorder“ OR “ophthalmic disorders“ OR “ophthalmic disease“ OR “ocular disease“ OR “eye disease“ OR “ophthalmic diseases“ OR “ocular diseases“ OR “eye diseases“ OR “ophthalmologic diseases“ OR “ophthalmologic disease“) AND (“Apnea“ OR “Apneas“ OR “Sleep Apnea Syndromes“ OR “Sleep Apnea Syndrome“ OR “Sleep Apnea“ OR “Sleep Apneas“ OR “Sleep Hypopnea“ OR “Sleep Hypopneas“ OR “Hypersomnia with Periodic Respiration“ OR “Mixed Central and Obstructive Sleep Apnea“ OR “Mixed Sleep Apnea“ OR “Mixed Sleep Apneas“ OR “Sleep-Disordered Breathing“ OR “Sleep Disordered Breathing“ OR “Obstructive Sleep Apnea” OR “Obstructive Sleep Apnea Syndrome” OR “Obstructive Sleep Apneas” OR “OSAHS” OR “Upper Airway Resistance Sleep Apnea Syndrome”) |
| GOOGLE SCHOLAR | ("floppy eyelid syndrome" OR "floppy eyelid") AND ("Apnea") |
| PROQUEST | NOFT(“floppy eyelid syndrome” OR “floppy eyelid” OR “eyelid laxity” OR “lax eyelid” OR “FES” OR ”ocular manifestations” OR “Eye manifestations“ OR “Eye manifestation“ OR “ophthalmic manifestations“ OR “ophthalmic manifestation“ OR “ocular pathology“ OR “ocular disorders“ OR “ocular disorder“ OR “eye disorders“ OR “eye disorder“ OR “ophthalmic disorder“ OR “ophthalmic disorders“ OR “ophthalmic disease“ OR “ocular disease“ OR “eye disease“ OR “ophthalmic diseases“ OR “ocular diseases“ OR “eye diseases“ OR “ophthalmologic diseases“ OR “ophthalmologic disease“) AND NOFT(“Apnea“ OR “Apneas“ OR “Sleep Apnea Syndromes“ OR “Sleep Apnea Syndrome“ OR “Sleep Apnea“ OR “Sleep Apneas“ OR “Sleep Hypopnea“ OR “Sleep Hypopneas“ OR “Hypersomnia with Periodic Respiration“ OR “Mixed Central and Obstructive Sleep Apnea“ OR “Mixed Sleep Apnea“ OR “Mixed Sleep Apneas“ OR “Sleep-Disordered Breathing“ OR “Sleep Disordered Breathing“ OR “Obstructive Sleep Apnea” OR “Obstructive Sleep Apnea Syndrome” OR “Obstructive Sleep Apneas” OR “OSAHS” OR “Upper Airway Resistance Sleep Apnea Syndrome”) |
