# Supplementary material for: Relationship Between Floppy Eyelid Syndrome and Obstructive Sleep Apnea Syndrome: An Umbrella Review
Source: J Ophthalmol. 2026 Jul 14;2026:6084912. doi: 10.1155/joph/6084912 (PMC13366405; doi:10.1155/joph/6084912)
Supplement: Supplementary file 2 — Supporting Information 2 Appendix 2—List of excluded full‐text articles and reasons for exclusion, cited in Section 3.1. [file JOPH-2026-6084912-s002.docx]

**Appendix 2 –** Excluded articles and reasons for exclusion (n=2).

**Author, year Reason for exclusion**

(M Santos and RJ Hofmann., 2017) 1

( Ezra et Al., 2010) 1

1) Literature reviews, case reports, personal opinions, letters, posters, and conference abstracts
